# Major depressive episode in the elderly. Use of maintenance ECT: a case report.

**DOI:** 10.1192/j.eurpsy.2024.1330

**Published:** 2024-08-27

**Authors:** A. Izquierdo De La Puente, P. del Sol Calderón, R. Fernandez Fernandez, M. Garcia Moreno

**Affiliations:** ^1^Psychiatry, Hospital Universitario Puerta de Hierro de Majadahonda, Majadahonda; ^2^Psychiatry, Hospital Universitario Infanta Cristina, Parla, Spain

## Abstract

**Introduction:**

We present the case of an elderly patient with a severe depressive episode who, in order to maintain psychopathological stabilisation, receives ECT on an outpatient basis.

**Objectives:**

The objective is to briefly review the use of ECT as a maintenance treatment for severe depression in the elderly.

**Methods:**

Patient aged 76 years, multipathological, with a history of hypertension, DM and LBP. Femoral head fracture, myelodysplastic syndrome, severe osteoporosis with vertebral crushing, requiring rescue treatment with tramadol, and renal failure.

She came for consultation, reporting depressive symptoms of months’ duration, together with delusions of ruin and nihilism. Despite antidepressant and stabilising treatment with duloxetine at daily doses of 120mg, extended-release quetiapine 600mg, lorazepam 2.5mg and mirtazapine 45mg, the patient began to show negative behaviour towards accepting food, clinophilic behaviour and abandonment, which led to her being admitted to the short-term hospitalisation unit.

**Results:**

Due to the severity of the depressive symptomatology, it was decided to start ECT, administering a total of 12 sessions, which were effective, and outpatient follow-up was resumed. However, after a week, the patient again began to show marked apathy and abulia, as well as complete anorexia lasting more than 24 hours, which led to a new admission. It was then that it was decided to maintain the ECT treatment, on an outpatient basis, as maintenance treatment, together with pharmacological treatment.

**Conclusions:**

ECT is indicated in severe depression, with or without psychotic symptoms, with malnutrition and organic pathology. According to studies, it has a beneficial response of more than 60%. However, the rate of receiving depressive symptomatology in a severe episode is high, despite ECT, so studies and clinical practice recommend maintenance ECT. It is usual to start with weekly sessions, and progressively space them out to maintain the minimum that guarantees stability.

**Disclosure of Interest:**

None Declared

